# Physiological condition of Eastern Baltic cod, *Gadus morhua*, infected with the parasitic nematode *Contracaecum osculatum*

**DOI:** 10.1093/conphys/coaa093

**Published:** 2020-09-22

**Authors:** Marie Plambech Ryberg, Peter V Skov, Niccolò Vendramin, Kurt Buchmann, Anders Nielsen, Jane W Behrens

**Affiliations:** 1 National Institute of Aquatic Resources, Technical University of Denmark (DTU Aqua), Kemitorvet 201, Kgs. Lyngby 2800, Denmark; 2 National Institute of Aquatic Resources, Technical University of Denmark (DTU Aqua), Willemoesvej 2, Hirtshals 9850, Denmark; 3 Department of Veterinary and Animal Sciences, Faculty of Health and Medical Sciences, University of Copenhagen, Stigbøjlen 7, Frederiksberg 1870, Denmark

**Keywords:** Compromised liver function, liver worm, parasites, energetic cost, nutritional condition, Eastern Baltic cod

## Abstract

Establishing relationships between parasite infection and physiological condition of the host can be difficult and therefore are often neglected when describing factors causing population declines. Using the parasite–host system between the parasitic nematode *Contracaecum osculatum* and the Eastern Baltic cod *Gadus morhua*, we here shed new light on how parasite load may relate to the physiological condition of a transport host. The Eastern Baltic cod is in distress, with declining nutritional conditions, disappearance of the larger fish, high natural mortality and no signs of recovery of the population. During the latest decade, high infection levels with *C. osculatum* have been observed in fish in the central and southern parts of the Baltic Sea. We investigated the aerobic performance, nutritional condition, organ masses, and plasma and proximate body composition of wild naturally infected *G. morhua* in relation to infection density with *C. osculatum*. Fish with high infection densities of *C. osculatum* had (i) decreased nutritional condition, (ii) depressed energy turnover as evidenced by reduced standard metabolic rate, (iii) reduction in the digestive organ masses, and alongside (iv) changes in the plasma, body and liver composition, and fish energy source. The significantly reduced albumin to globulin ratio in highly infected *G. morhua* suggests that the fish suffer from a chronic liver disease. Furthermore, fish with high infection loads had the lowest Fulton’s condition factor. Yet, it remains unknown whether our results steam from a direct effect of *C. osculatum*, or because *G. morhua* in an already compromised nutritional state are more susceptible towards the parasite. Nevertheless, impairment of the physiological condition can lead to reduced swimming performance, compromising foraging success while augmenting the risk of predation, potentially leading to an increase in the natural mortality of the host. We hence argue that fish–parasite interactions must not be neglected when implementing and refining strategies to rebuild deteriorating populations.

## Teaser

The nutritional condition; the energy turnover; the body, liver and plasma composition; and the digestive organ masses were evaluated in relation to varying infection densities with liver worm in Eastern Baltic cod. We show that fish with high infection load with this parasitic nematode have severely compromised physiological condition and poor health status.

## Introduction

Parasitism is one of the most common animal lifestyles and can impact ecosystem functioning by affecting food-web stability, interaction strength and energy flow in both terrestrial and aquatic ecosystems (Marcogliese, 2004; Kuris *et al*., 2008; Lafferty *et al*., 2008; Hatcher *et al*., 2014). At the level of the individual, parasites can cause adverse effects on the performance capacity of the host (McElroy and de Buron, 2014), e.g. by changing plasma protein and hormone levels (Akinyi *et al.*, 2019; O’Dwyer *et al.*, 2019), reducing aerobic and locomotor performances (Umberger *et al*., 2013; Hahn *et al*., 2018) and depleting energy reserves (Ferrer-Maza *et al.*, 2016). Together, this shapes the physiological condition of an infected individual, and impairment may lead to reduced growth and increased mortality (Marcogliese, 2004; Khan, 2005; Behrens *et al*., 2014). For trophically transmitted parasites, such effects on transport hosts (transport hosts having only sexually immature stages of the parasite, and with no development of the parasite occurring while hosting it; Rohde, 2002) can either be a result of accidental side-effects associated with the infections (Hafer and Milinski, 2016) or host manipulations (McElroy and de Buron, 2014) or maybe even a combination of both (Heil, 2016). Yet, irrespectively of the reason, it makes the host more vulnerable to predators, increasing the probability of the parasite to reach its final host (Heil, 2016).

Marine fish are hosts to a high diversity of parasitic organisms (Marcogliese, 2002; Rohde, 2002), and parasite-induced impairment of the physiological condition has been suggested to reduce fish stock productivity, leading to declining catches of both freshwater and marine fish populations (Lloret *et al.*, 2012). However, establishing causality between parasite infection and physiological condition of the host can be difficult, and the mechanisms underlying parasite-altered host fitness remain largely unknown (Lloret *et al.*, 2012; McElroy and de Buron, 2014).

Here, we use the host–parasite system between the third stage larvae liver worm *Contracaecum osculatum* (Zuo *et al.*, 2018) and Eastern Baltic cod *Gadus morhua* as a case study to investigate the physiological performance of wild fish with high parasite load. The Eastern Baltic cod stock has exhibited a decline in the nutritional condition during the past 20 years, an event that has occurred alongside deteriorating oxygen conditions and reduced prey abundance, now leaving the fish historically malnourished and growth impaired (Eero *et al*., 2015; Casini *et al*., 2016b; Hüssy *et al*., 2018; Neuenfeldt *et al*., 2020). This has challenged the management of the stock which at present shows no signs of recovery, and with high natural mortality (ICES, 2019) and a fishing ban introduced in 2019. The grey seal *Halichoerus grypus* population has been severely reduced due to hunting and breeding problems between the 1960s and the 1990s where after recovery slowly began (Harding *et al.*, 2007). Concurrent with the recovery of *H. grypus*, an increase in infections with the trophically transmitted *C. osculatum* that parasitizes the liver of cod has also been observed in *G. morhua* in the central and eastern Baltic Sea since the early 2010s (Haarder *et al*., 2014; Nadolna and Podolska, 2014). This has coincided with even further deterioration of the health status and stock productivity of the fish (Eero *et al*., 2012, 2015). *H. grypus* is the main final host of this parasite while cod act as the last transport host in the life cycle (Koie and Fagerholm, 1995; Nadolna-Ałtyn *et al*., 2018; Zuo *et al*., 2018).

Field investigations have shown that infection intensity with *C. osculatum* in *G. morhua* coincides with poor nutritional status, and that more Westerly and Northwesterly cod stocks with little or no *C. osculatum* are in better nutritional condition (Horbowy *et al*., 2016; Sokolova *et al*., 2018). This parasite migrates to the liver of the cod following ingestion via smaller infected prey, e.g. sprat *Sprattus sprattus* (Zuo *et al*., 2016; Nadolna-Ałtyn *et al*., 2018), where it accumulates over time, resulting in a larger parasite burden in older fish (Horbowy *et al*., 2016; Zuo *et al*., 2016). The liver is responsible for nutrient assimilation, bile production, maintenance of metabolic homeostasis and protein synthesis and also serves as an energy reserve and breeding capital for the fish (Hinton *et al*., 2017). It is thus intuitive to think that a high liver parasite burden leads to reduced function of the organ with negative effects on the nutritional condition of the infected individual. Yet, disentangling potential effects of parasites on their hosts from effects arising in the wake of unfavourable abiotic and food conditions demands an interdisciplinary approach combining field and laboratory studies, and expert parasitologists, physiologists and biologists (McElroy and de Buron, 2014).

To elucidate how high parasite load may relate to the physiological condition of wild fish, we here investigated aerobic performance, nutritional condition, mass of selected organs and plasma and proximate body composition of wild, naturally infected *G. morhua* in relation to infection density with *C. osculatum*.

## Materials and methods

### Pilot study

The number of *C. osculatum* in cod livers increases with the length of the fish (Nadolna and Podolska, 2014; Horbowy *et al*., 2016). In 2017, we therefore conducted a pilot study to identify the length interval of *G. morhua* needed to obtain fish samples with sufficient variability in infection intensity to make a solid study design. More specifically, wild and naturally infected *G. morhua* (*n* = 86) were captured by trawl East of Bornholm and used to assess the correlations between body mass (BM), total length (TL), liver mass (LM), gender and number of nematodes in the liver. A length range between 30 and 53 cm provided sufficient high variability in infection intensity of nematodes in the livers of the fish. Fish for experiments (see below) were consequently selected to cover this length range if possible.

### Experimental animals

Wild naturally infected Eastern Baltic cod (*n* = 152) were captured by trawl East of Bornholm between 2017 and 2019 (Table 1). Cod were either used live for respirometry or sampling was made directly on dead fish for investigations of body composition and organ sizes. For the latter investigations, fish were killed and frozen at −20°C immediately after trawling, and transported to DTU Aqua, Lyngby, for later analysis (Table 1). For fish used live in experiments (Table 1), trawling time never exceeded 20 min in order to minimize stress and damage to the fish. Live cod were acclimated for 2 weeks in captivity at Bornholm’s Salmon Hatchery before being transferred to the fish-holding facilities at DTU Aqua, Lyngby. Here, they were held in two circular tanks (2000 L each) with air-saturated recirculated water (10°C, 10‰ salinity, photoperiod of 8:16 light: dark, with a half-hour period of sunrise and sunset) and allowed 3 weeks of acclimation before initiation of experiments. Fish were fed three times weekly with cooked blue mussels corresponding to ~2% of their BM. All experiments were carried out according to the animal welfare regulations of the Technical University of Denmark and EU directive 2010/63/EU for animal experiments. Ethical permit 2017-15-0201-01282 from the Danish Animal Ethics Committee covered all experiments reported here.

**Table 1:** Overview of fish within all six assessments. *n* = number of fish, TL = total length, BW = body weight, LM = liver mass, prevalence: percentage of infected fish in the sample, and intensity of infection: mean number of parasites per liver, including only infected individuals. The numbers in brackets represent ranges of variables. All numbers are mean ± SE

Assessment	*n*	TL (cm)	BW (g)	LM (g)	Prevalence (%)	Intensity
Nutritional condition	152	39 ± 0.4 (29–53)	510 ± 14.9 (209–1098)	21 ± 1.0 (4–80)	89	32 ± 2.7 (0–203)
Aerobic performance and plasma composition	60	42 ± 0.5 (34–53)	572 ± 22.2 (260–1077)	19 ± 1.4 (4–57)	93	46 ± 5.3 (0–203)
Proximate composition of fish and liver	33	40 ± 1.0 (29–53)	532 ± 41.7 (212–1098)	25 ± 3.3 (6–80)	85	23 ± 4.3 (0–104)
Organ size	59	36 ± 0.5 (28–45)	434 ± 16.7 (209–780)	20 ± 9.2 (5–41)	86	21 ± 2.5 (0–72)

### Recovery of nematodes from cod livers

All livers from fish used in the present study were dissected out and frozen separately (−20°C) before they were analysed for the presence of nematodes. Individual livers, except those used for lipid and energy analysis, were placed in a plastic bag (200 × 400 × 0.07 mm) and compressed between two glass plates (15 × 15 × 1 cm) to a thickness of 1 mm by the addition of gentle pressure to the plates (Buchmann, 2007). Livers were subsequently examined under a Leica stereo microscope (6.3–40× magnification) (Leica Microsystems Germany), and detected nematodes were categorized as either small (<1-cm body length) or large (>1-cm body length). To minimize oxidation processes and tissue breakdown in livers dedicated to lipid analysis, individual defrosted livers (*n* = 33) were placed on glass petri dishes kept on ice. Single nematodes were manually removed, using a tweezer, and the total number for each liver recorded and subsequently preserved in 70% ethanol. For all examined livers, nematode species identification was based on morphometric characteristics of the caudal and cephalic ends according to Fagerholm (Koie and Fagerholm, 1995). To compensate for differences in the number of nematodes related to liver size, infection density was calculated as the number of nematodes per gram of liver tissue (i.e. liver tissue = wet weight of the liver minus total weight of nematodes), rather than the total number of nematodes per liver.

### Nutritional condition

The association between infection density with nematodes and the nutritional condition of all cod included in this study (*n* = 152) was analysed by calculating the Fulton condition factor:

$$\mathrm{Fulton}\ \mathrm{condition}\ \mathrm{factor}=\left(\frac{\mathrm{BM}}{{\mathrm{TL}}^3}\right)\ast 100$$

### Aerobic performance

To investigate potential associations between infections and the aerobic performance of the fish, we determined standard metabolic rate, maximum metabolic rate and aerobic metabolic scope (i.e. maximum metabolic rate minus standard metabolic rate) of cod (*n* = 60) with varying infection densities of nematodes. The standard metabolic rate represents the energy requirements of the individual at a resting, non-digesting state; maximum metabolic rate represents the maximum aerobic performance (Chabot *et al*., 2016), while aerobic metabolic scope relates to the ability to perform aerobic work. Four static respirometers (6.6 L or 8.2 L, to accommodate for differences in TL of fish) were placed in a 250-L black tank supplied with a flow-through of aerated water from the same supply as the holding tanks (10°C, 10‰ salinity). To minimize disturbance from the neighbouring fish, non-transparent polyethylene plates were placed vertically between the respirometers, and to minimize disturbance from the outside, a curtain shielded the setup.

For standard metabolic rate determinations, oxygen consumption rates (mg O_2_ kg^−1^ h^−1^) were measured over a period of > 40 h, using intermittent-flow respirometry (Steffensen, 1989). Oxygen consumption rate was measured in 12-min loops consisting of a flush (420 s), a wait (60 s) and a measurement (240 s) period. To obtain maximum metabolic rate, individual fish were exposed to a chase protocol (intense continuous chasing for 2 min in a circular 300-L tank) and immediately thereafter (within maximally 2 min) placed in the respirometer, where the first oxygen consumption values were obtained rapidly, using measurement periods of 90 s, with 1 s of wait and without flushing. Measurements for maximum metabolic rate were terminated if oxygen saturation fell below 75% within the 90-s measurement period. Hereafter, oxygen consumption measurements continued in 12-min loops (as described above) for a minimum of 40 h (i.e. for standard metabolic rate determination).

The highest oxygen consumption measurement (i.e. maximum metabolic rate) occurred instantly after the chase protocol for 51 of the fish, whereas the maximum metabolic rate occurred later (following spontaneous activity inside the respirometer) for the remaining nine fish. For each fish, the standard metabolic rate was determined as the average of the 10% the lowest oxygen consumption (Chabot *et al.*, 2016) values, and aerobic metabolic scope was calculated as the difference between the maximum and standard metabolic rate. Background respiration was found by linear regression of the start and the final background measurements and subsequently subtracted from the corresponding oxygen consumption value. To eliminate potential effects of digestion on oxygen consumption, all cod were fasted 3–4 days prior to the experiment, the specific number of days being based on the cod gastric evacuation model made by Andersen, 2012. All cod were weighed upon initiation of the experiment. To assess for potential contributions from nematode oxygen consumption to the measured oxygen consumption of the cod, oxygen consumption (mg O_2_ kg^−1^ h^−1^) was determined on 85 live nematodes (0.001–0.012 g) over a 24-h period, using a 24-well glass microplate containing 5-mL wells (Loligo Systems) with 10°C and 10‰ salinity water.

### Organ size

To elucidate associations between infection density and size of selected organs, whole cod (*n* = 59) were thawed, and BM, TL, LM, gender and weight to the nearest gram of the stomach (empty), intestine (empty), pyloric caeca and heart were recorded.

### Plasma composition

To reveal potential associations between the nematodes and the function of the liver and the disease status of the fish, the haematological analysis was performed. Following respirometry, fish (*n* = 60) were stunned by a sharp blow to the head; blood was immediately sampled by caudal puncture with a lithium-heparinized 21-gauge hypodermic needle, and fish were euthanized by spinal transection. Blood samples were centrifuged at 1610*G* for 5 min, and the plasma fraction was stored at −18°C (Houston, 2002). Total blood protein content (g L^−1^) was determined using an ADVIA 1800 Clinical Chemistry System (Siemens), while the separation of plasma protein fractions into pre-albumin, albumin and the globulins (alpha-1, alpha-2, beta-1, beta-2 and gamma) was done using capillary electrophoresis (MINICAP PROTEIN 6, Sebia, Lisses). A/G ratios were calculated by dividing individual plasma albumin and globulins values.

### Proximate composition and energy content

In order to examine changes in body composition and energy content of cod in relation to different infection densities, 33 whole cod (livers removed and with empty stomachs) were individually autoclaved and homogenized (Table 1). Crude protein (N^*^ 6.25) content of the fish, and crude lipid content of fish and livers, were determined using the Kjeldahl (Foss Kjeltec 2200, Hillerød, Denmark) and the Bligh and Dyer methods (Bligh and Dyer, 1959), respectively. To obtain dry matter and water content of the fish and liver, samples were dried for 24 h at 105°C, and weight loss was determined (Memmert UN110, Büchenbach, Germany). Ash content determinations were based on weight loss after incineration of samples for 6 h at 550°C in a muffle furnace (Heraeus Instruments K1252, Hanau, Germany) (Obirikorang *et al*., 2016), and glycogen content of the fish was calculated as the difference between the initial dry weight and the sum of the crude protein, fat and ash weights (Saint-Paul, 1984). The energy density of dry cod tissue was determined from dried tissue samples combusted in a Parr 6300 bomb calorimeter and subsequently converted to energy density per gram of wet BM (kJ g^−1^) (Schloesser and Fabrizio, 2017).

### Data handling and analysis

Prior to infection density calculations, the total weight of nematodes retrieved from individual livers was subtracted from the measured LM, small nematodes being assigned a weight of 0.004 g and large nematodes 0.009 g, based on the weight range of nematodes used in respirometry. Oxygen consumption measurements with *R*^2^ < 0.95 (in total <0.8%) were excluded from the analysis. Two of the 60 fish used in respirometry never entered into a resting state (judged by observations of the raw oxygen consumption data) and were hence excluded from the dataset.

Log-linear Gaussian models were used to describe the associations between infection density, TL and gender in all assessed variables except in the analysis of changes in organ size where power function models were used instead. TL was included to account for the accumulation of nematodes in the liver over time (i.e. with the increasing length of the fish) (Nadolna and Podolska, 2014; Horbowy *et al*., 2016), and gender was included to test for any potential differences between males and females. TL was not included in the analysis of standard metabolic rate, maximum metabolic rate and aerobic metabolic scope as these were modelled as the mass-specific oxygen consumption because the scaling exponent for the relationship between oxygen consumption and body mass of the fish was 1. Likewise, TL was not included in the analysis of the Fulton condition factor as TL is included in this parameter. For the analysis of fish body composition, the effect of infection density on all the performed analyses was carried out together with analysis of changes in the whole-body energy content, as well as the redistribution between protein, lipid and glycogen and water within the fish. To test the robustness of the results (due to high variation in infection density), we divided fish into three groups of infection densities, non-, medium- and high-infected, to test for significant difference between these three groups for selected parameters.

All statistical tests were conducted in R (R Core Team, 2016). Before model fitting, collinearity between explanatory variables was assessed by using variance inflation factors (VIF) (Zuur *et al*., 2009). No variables were excluded from the analysis due to collinearity (Tables S1 and S2). Model selection was performed using a stepwise backward selection routine based on the likelihood ratio test for each of the variables included and excluded in the models. The model assumptions of normality and independence were subsequently validated by visual inspection of model residuals (Figs S1–S12). ANOVA and post hoc (Tukey HSD) were used for the test of robustness.

## Results

**Figure 1:**
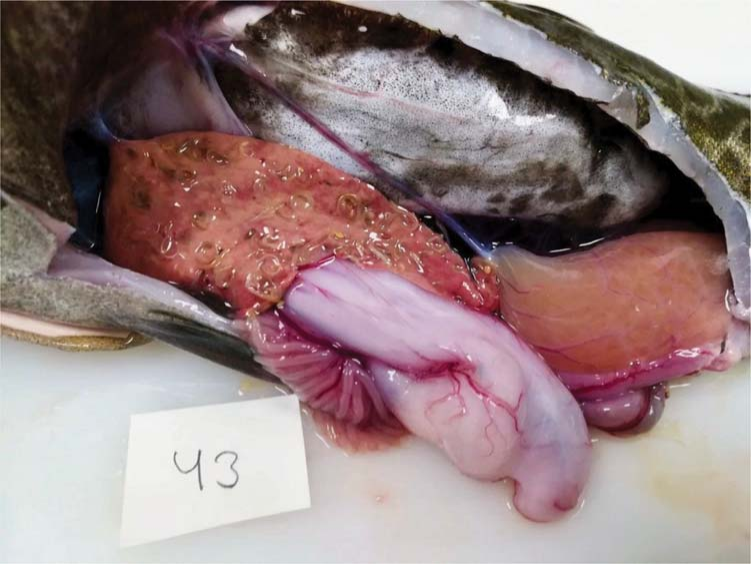
An example of a *G. morhua* with a liver having a high density of *C. osculatum* (19 nematodes per gram liver tissue), some visible on the surface of the organ, others hidden in the liver parenchyma

A total of 4309 nematodes were recovered from the 152 livers examined, all belonging to the species *C. osculatum*. The mean and range of infection density were 2 (±SE 0.3) and 0–22 nematodes per gram liver, respectively. Upon retrieval of the livers from the fish for recovery of nematodes, it was noticed that for the 12 most heavily infected livers (all with infection density above six nematodes per gram liver; Fig. 1), the organ seemingly was losing its structure/integrity, and ‘melted’ upon removal from the body cavity of the fish.

### Nutritional condition

**Figure 2:**
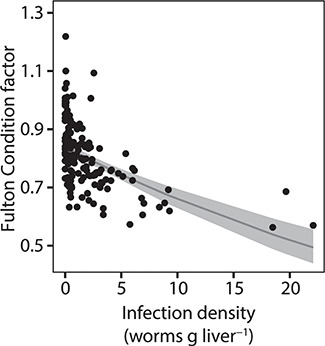
Fulton condition factor of cod (*n* = 152) with varying degrees of infection densities with *C. osculatum*. The thin grey line describes the model fit, a significant negative association between infection density and the Fulton condition factor. The 95% confidence interval is represented by the grey area

The Fulton condition factor of cod decreased significantly (GLM: *n* = 152, SE = 0.003, *t* = −8.1, *P* < 0.001) with increasing infection density (Fig. 2, Tables 2 and 3).

**Table 2:** Symbols reflect the estimates of the final models (SE in brackets): *α* = infection density (INF), *β* = intercept, *γ* = gender (estimate for female), *μ* = length and *λ* = total energy. - = variable not significant in the model, and empty columns = variable was not included in the full model. Units of parameters: 1 = mg O_2_ kg^−1^ h^−1^, 2 = g, 3 = g  L^−1^, 4 = %, 5 = kJ g^−1^ and 6 = g g liver^−1^. SMR = standard metabolic rate. Asterisks indicate the significance level of the estimated parameters (^*^P < 0.05, *P* < ^**^0.01, ^***^P < 0.001). *R*^2^ indicates how much of the variation of data each model explains. All reported estimated model parameters are on log scale except for organ size where estimates are on a log-10 scale

Assessment	Parameter	*α* (INF)	*β* (intercept)	*γ* (gender)	*μ* (length)	*λ* (total energy)	*R* ^2^
Nutritional condition	Fulton condition	−0.02 (0.003)^***^	−0.17 (0.01)				0.30
Aerobic performance	SMR^1^	−0.01 (0.003)^**^	3.98 (0.02)	-			0.14
Organ size	Pyloric caeca^2^	−0.02 (0.01)^*^	−3.44 (0.55)	-	2.60 (0.36)^***^		0.47
	Intestine^2^	−0.02 (0.01)^*^	−2.94 (0.54)	0.08 (0.03)^*^	2.22 (0.35)^***^		0.43
Plasma composition	Total protein^3^	−0.01 (0.005)^**^	3.49 (0.03)	-	-		0.13
	Globulins^4^	0.01 (0.003)^***^	4.35 (0.02)	-	-		0.19
	A/G	−0.16 (0.03)^***^	−1.5 (0.17)	-	-		0.33
	Pre-albumin^4^	−0.08 (0.02)^***^	2.35 (1.42)	0.64 (0.01)^**^	0.06 (0.03)^*^		0.21
	Gamma^4^	0.02 (0.008)^*^	2.16 (0.04)	-	-		0.07
	Albumin^4^	−0.15 (0.03)^***^	2.84 (0.16)	-	-		0.33
Proximate composition of fish	Total energy^5^	−0.03 (0.01)^**^	1.28 (0.01)	-	-		0.18
	Water^4^	0.003 (0.001)^*^	4.39 (0.002)	-	-		0.14
	Protein^5^	−0.04 (0.01)^**^	1.03 (0.02)	-	-		0.25
	Glycogen^5^	0.05 (0.01)^**^	0.36 (0.02)	-	-		0.20
	Ash^4^	0.04 (0.01)^*^	1.25 (0.02)	-	-		0.14
	Dry matter^4^	−0.01 (0.006)^*^	2.95 (0.01)	-	-		0.14
	Protein^5^		−0.43 (0.09)	-	-	0.40 (0.02)^***^	0.89
	Glycogen^5^		1.71 (0.20)	-	-	−0.38 (0.06)^***^	0.57
Proximate composition of liver	Lipid^6^	−0.11 (0.03)^***^	3.59 (0.24)	0.15 (0.07)^*^	-		0.41
	Water^4^	0.10 (0.03)^***^	3.76 (0.06)	−0.15 (0.07)^*^	-		0.37
	Dry mat^4^	−0.10 (0.03)^***^	4.00 (0.06)	0.15 (0.07)^*^	-		0.41
	Ash^4^	0.10 (0.03)^**^	−0.49 (0.06)	-	-		0.18

**Table 3:** Results of post hoc analysis Tukey HSD test for eight variables where infection density is divided into three groups. For the first four variables, infection density (number of nematodes per gram liver tissue) in each group is non = 0, medium = 4 and high>4, and for the four latter variables, infection density in each group is non = 0, medium = 2 and high>2 as a result of different range in infection densities between the two batches of fish. Units of parameters: 1 = mg O_2_ kg^−1^ h^−1^, 2 = g L^−1^, 3 = kJ g^−1^, 4 = % and 5 = g g liver^−1^. *P* value = overall significance level between the groups, letters = groups that do not differ statistically from each other. ^*^ visualizes how group b or c differs significantly from a group where ^*^P < 0.05, ^**^P < 0.01 and ^***^P < 0.001. *n* represents the number of fish within each infection group (non, medium, high). SMR = standard metabolic rate. All numbers are mean ± SE

Parameter	Non	Medium	High	*P* value	*n*
Fulton condition	0.88 ± 0.2(a)	0.81 ± 0.1(b^**^)	0.68 ± 0.2(c^***^)	<0.001	(17, 115, 20)
SMR^1^	56.3 ± 0.5(a)	54.6 ± 0.2(a)	44.7 ± 0.3(b^**^)	0.002	(4, 39, 15)
Total protein^2^	32.1 ± 0.6(ab)	32.4 ± 0.2(a)	27.8 ± 0.3(b^*^)	0.02	(4, 39, 15)
A/G	0.33 ± 0.9(a)	0.18 ± 0.4(a)	0.04 ± 1.3(b^**^)	<0.001	(4, 39, 15)
Total energy^3^	3.67 ± 0.5(a)	3.49 ± 0.2(ab)	3.32 ± 0.4(b^*^)	0.05	(5, 21, 7)
Water fish^4^	80.2 ± 0.4(a)	81.2 ± 0.2(ab)	81.4 ± 0.4(b^*^)	0.03	(5, 21, 7)
Lipid liver^5^	0.58 ± 0.5(a)	0.45 ± 0.3(a)	0.30.1 ± 0.6(b^**^)	0.03	(5, 21, 7)
Water liver^4^	36.6 ± 0.5(a)	44.2 ± 0.3(ab)	54.0 ± 0.5(b^**^)	0.02	(5, 21, 7)

### Aerobic performance

**Figure 3:**
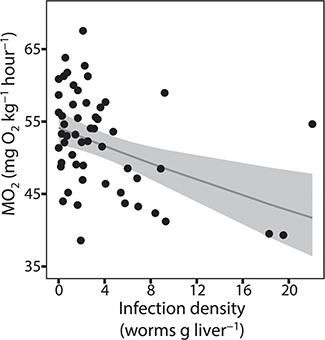
The standard metabolic rate (MO_2_, in mg O_2_ kg^−1^ h^−1^) of *G. morhua* (*n* = 58) with varying degrees of infection densities with *C. osculatum*. The thin grey line describes the association between infection density and standard metabolic rate, revealing a significant negative correlation. The grey boxes are 95% confidence intervals

The standard metabolic rate decreased significantly (GLM: *n* = 58, SE = 0.003, *t* = −3.2, *P* = 0.002) with increasing infection density (Fig. 3, Tables 2 and 3). In contrast, the maximum metabolic rate was not associated with changes in infection density and aerobic metabolic scope, and gender was not significant in any of the three cases. The oxygen consumption of *C. osculatum* was inconsiderable (mean ± SE; 0.0002 ± 2.2E^−05^, mg O_2_ kg^−1^ h^−1^) and thus negligible for the measured oxygen consumption of the cod.

### Organ size

The mass of intestine and pyloric caeca increased significantly (GLM: intestine, *n* = 58, SE = 0.350, *t* = 6.3, *P* < 0.001 and pyloric caeca, *n* = 58, SE = 0.357, *t* = 7.3, *P* < 0.001) with the length of the fish but decreased significantly with increasing infection density (GLM: intestine, *n* = 58, SE = 0.012, *t* = −2.1, *P* = 0.03 & pyloric caeca, *n* = 58, SE = 0.011, *t* = −2.2, *P* = 0.03). Females had larger intestines compared to males, but gender could not explain any variation found in the weight of pyloric caeca (Table 2).

### Plasma composition

**Figure 4:**
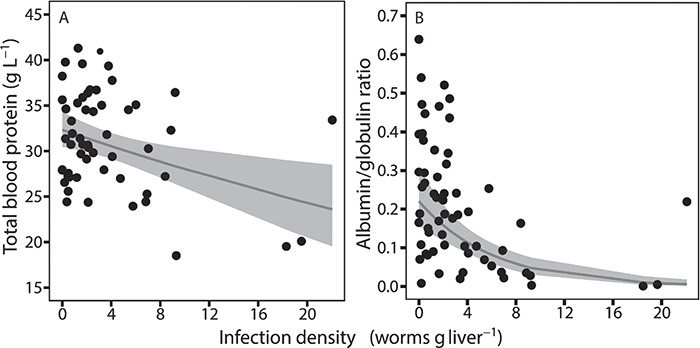
(**A**) Total blood protein (g L^−1^) and (**B**) albumin/globulin ratio in *G. morhua* (*n* = 60) in relation to varying degrees of infection densities with *C. osculatum*. Both parameters decreased significantly with increasing infection density, as described by the thin grey lines. The grey boxes are 95% confidence intervals

Total protein in the plasma decreased significantly (GLM: *n* = 60, SE = 0.005, *t* = −3.1, *P* = 0.01; Fig. 4A) with increasing infection density, as did the albumin to globulin ratio (GLM: *n* = 60, SE = 0.030, *t* = −5.4, *P* < 0.001; Fig. 4B) (Tables 2 and 3). Albumin decreased significantly while the sum of globulins and gamma-globulins alone increased significantly with increasing infection density (Table 2). These changes in protein fractions of the plasma were reflected by the highly significant decrease in albumin to globulin ratio. Pre-albumin, which is not a part of the albumin to globulin ratio, decreased significantly with increasing infection density (Table 2). On the contrary, there was no change in alpha-1, alpha-2 and beta-1-2 with increasing infection density. Gender and TL were only significant in the analysis of pre-albumin (Table 2).

### Proximate composition and energy content

**Figure 5:**
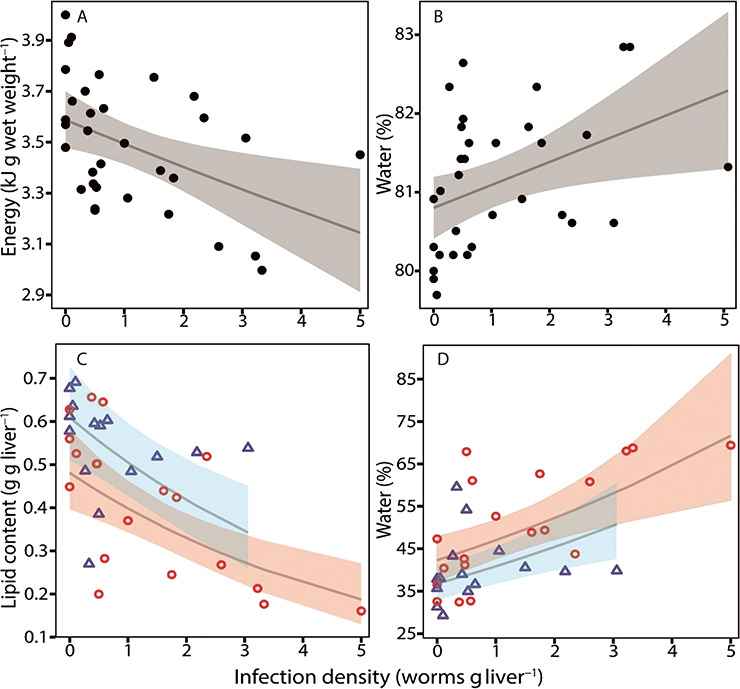
(**A**) Total energy content (kJ g wet-weight^−1^) of the whole fish excluding the liver, (**B**) water content (%) of the whole fish, (**C**) lipid content of the liver (g g liver^−1^) and (**D**) water content of the liver (%), all in relation to changes in infection density as described by the thin grey lines. Both the total energy of the fish and lipid content of the liver decreased significantly with increasing infection density while water content increased significantly in both the fish and the liver with increasing infection density. In C and D, colours and symbols represent: blue and Δ = female, red and o = male. Grey, blue and red areas represent 95% confidence intervals.

Overall, the body composition of the fish changed with increasing infection density. More specifically, total energy (GLM: *n* = 33, SE = 0.009, *t* = −2.8, *P* = 0.006; Fig. 5A) and protein content decreased significantly with increasing infection density (Table 2), while water (GLM: *n* = 33, SE = 0.001, *t* = 2.5, *P* = 0.01; Fig. 5B) and glycogen content increased significantly (Table 2).

**Figure 6:**
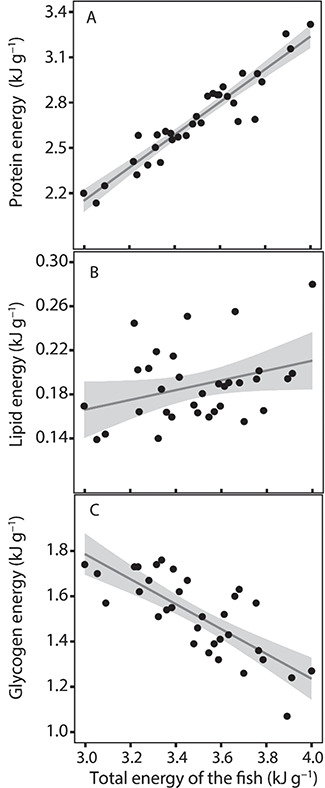
Proportions of the fish energy source coming from (**A**) protein energy (kJ g^−1^), (**B**) lipid energy (kJ g^−1^) and (**C**) glycogen energy (kJ g^−1^), all in relation to the total energy of the fish (*n* = 33). As described by the thin grey lines, the proportion of energy in the fish coming from protein increased significantly with increasing energy of the fish, and likewise for lipid energy (though not significantly), whereas the amount of energy coming from glycogen sources decreased with the energy of the fish. The grey areas represent a 95% confidence interval

The decrease in total energy content of the fish was explained by a significant change in the source of energy: a significant increase in protein (GLM: *n* = 33, SE = 0.025, *t* = 16.1, *P* < 0.001; Fig. 6A) and a slight increase in lipid energy, and a significant decrease in glycogen energy (GLM: *n* = 33, SE = 0.057, *t* = −6.6, *P* < 0.001; Fig. 6C) with an increase in the total energy of the fish (Fig 6, Table 2).

The total lipid energy of the fish did not change with changing infection density, and gender could not be associated with the observed changes in the body composition of the fish. Liver lipid content (GLM: *n* = 33, SE = 0.116, *t* = 2.1, *P* < 0.001; Fig. 5C) and dry matter decreased significantly with increasing infection density, while ash and water content (GLM: *n* = 33, SE = 0.074, *t* = −2.0, *P* = 0.001; Fig. 5D) significantly increased with increasing infection density. Lipid, water and dry matter contents of the liver differed significantly between males and females, as livers from females contained more lipid and less water and less dry matter content compared to livers from males (Table 2).

### Test of robustness

Fulton condition factor decreased significantly between the non-, medium- and high-infected groups with the lowest value found in the high infected group (Table 3). The standard metabolic rate, A/G ratio and lipid content of the liver decreased significantly in the high-infected groups compared to the non- and medium-infected groups (Table 3). Total energy and water content of the fish and the liver did not differ significantly between the high and medium groups but only between the non- and high-infected groups (Table 3). Total protein in the plasma did not differ significantly between non- and high-infected groups, only between the high- and the medium-infected groups (Table 3).

## Discussion

Using an integrative approach, we show that wild naturally infected *G. morhua* with heavy infections with the parasitic *C. osculatum* have impaired nutritional condition, reduced functionality of the digestive system, as indicated by the observed reduction in digestive organ masses (intestine and pyloric caeca), reduced baseline metabolism and changes in the body and plasma composition and fish energy source. Notably, fish with high infection densities had decreased plasma albumin and increased globulin levels, resulting in reduced albumin to globulin ratio.

Synthesized in the liver, albumin is the main protein of the blood plasma. It has several functions, e.g. maintaining osmotic pressure, capillary permeability and transport and metabolism of an extraordinarily diverse range of molecules (McDonald and Milligan, 1992; Garcia-Martinez *et al.*, 2013). In humans, reduced albumin to globulin ratios (driven by reduced albumin and increased globulins) are seen in individuals with chronic liver diseases associated with parenchymal damage, such as cirrhosis and liver cancer. Here, the increased globulin levels are caused by alternations in the gamma fraction (synthesized in lymphatic tissues), with alpha and beta globulins remaining stable (Teloh, 1978; Suh *et al.*, 2014). Changes in plasma protein composition also occur in diseased fish, where the albumin to globulin ratio has been used to reveal the physiological effects of specific pathogens (Aydin *et al.*, 2001; Osmani *et al.*, 2009). Notably, for some fish species, gamma globulins are considered to represent the complex nature of parasitic nematode antigenicity (Meade and Harvey, 1969), which is in agreement with the present response of *G. morhua* to a parasitic nematode, where the gamma fraction of the globulins drives the observed increase in the plasma globulins. In support, in *G. morhua*, genes related to immune response are overall affected by infections with *C. osculatum* (Marnis *et al*., 2019). More specifically, expression of immune-related genes in *G. morhua* tends to be downregulated in the liver but upregulated in the spleen, suggestively due to local immune suppression in the liver caused by *C. osculatum* (Marnis *et al*., 2020). Taken together, we argue that the significant decrease in the albumin to globulin ratio (caused by concomitant changes in plasma albumin and gamma globulin) that occurs with increasing *C. osculatum* infection reveals that highly infected *G. morhua* suffer from a chronic pathological liver condition. This in turn probably impairs the organ functionality, likely due to extensive tissue damage by *C. osculatum* (Fig. 1) (Buchmann and Mehrdana, 2016).

**Figure 7:**
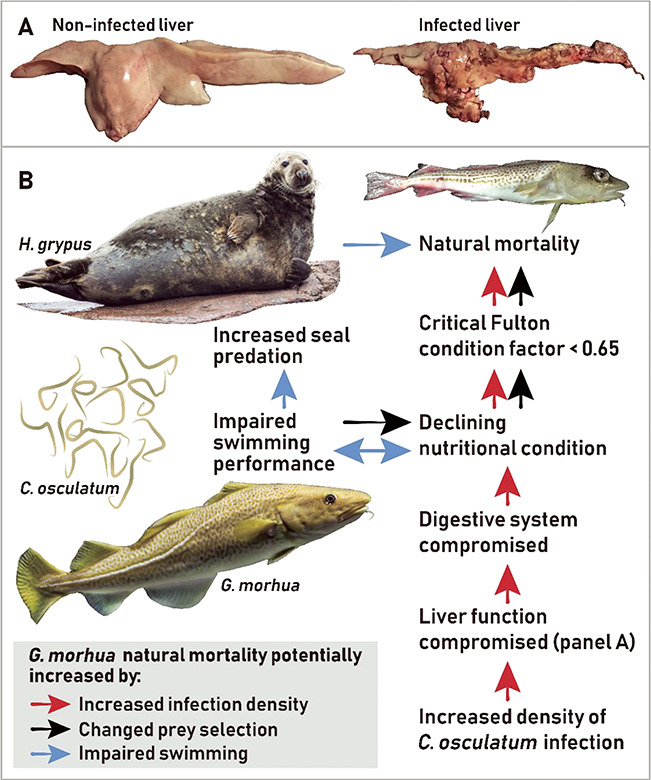
Schematic overview that summarizes possible mechanisms that suggestively can lead to increased natural mortality of *G. morhua* with high infection densities of *C. osculatum*, a parasitic nematode that infects the liver of the fish. Infections are associated with compromised function of the liver and digestive system that can lead to declining nutritional condition (red arrows) and potentially impaired swimming performance resulting in augmented susceptibility towards predation by seal (blue arrows) and changes in prey selection (black arrows). All mechanisms may lead to an increase in natural mortality. The critical Fulton condition factor where cod are considered dying is defined in Casini *et al.*, 2016a based on findings of Dutil and Lambert, 2000

Many parasites impose an energetic cost to their host (Lester, 1971; Östlund-Nilsson *et al*., 2005; Binning *et al*., 2013); yet, we saw reduced standard metabolic rate in heavily infected fish. Because standard metabolic rate represents the summated energy expenditure *in vivo*, pinpointing the specific cause for this with certainty is not possible. However, as the site of infection of this particular parasite is the liver, and because the specific site of infection often determines how the parasite affect its hosts (Lafferty and Shaw, 2013), we suggest that a main cause of the reduced standard metabolic rate is an impaired functionality of this accessory digestive organ, leading to a compromised digestive system. This would result in reduced efficiency in protein turnover, reflected in a loss of body protein, low plasma total protein and albumin, and decrease in body energy content and a shift in the composition of the body energy in fish as seen in the present study for *G. morhua* with high infection densities. Furthermore, as a large proportion of the maintenance costs are directed towards internal organs (Hulbert and Else, 2000). The observed decreased mass of intestinal tissue and pyloric caeca in highly infected fish may also partly explain the reduced standard metabolic rate. It is nevertheless important to note that the present results are derived from wild, naturally infected fish, with unknown feeding histories, potentially including periods of starvation. Starvation elicits a cascade of physiological responses, and many ectotherms (including fishes) and endotherms have been reported to reduce the mass of their gastrointestinal tissues to reduce energetic demands during starvation (McCue, 2010; Day *et al.*, 2014). Furthermore, during starvation, *G. morhua* initially exhausts its hepatic lipid and glycogen storage, and muscle glycogen, followed by mobilization of muscle protein (substituted by water) (Black and Love, 1986; Navarro and Gutiérrez, 1995). Thus, the reduced protein and lipid contents observed in the present study on *G. morhua* with high infection densities in some aspects resemble a starvation response. Yet, one aspect differs markedly; fish that have starved long enough to deplete their hepatic lipid and muscle glycogen resources rely on muscle protein as the main source of energy (Navarro and Gutiérrez, 1995), whereas in the present study, glycogen energy was found to constitute the main proportion of the fish energy source for the fish with the highest infection intensities. Based on this, it seems plausible that high infection densities, and not starvation, are the main driver of the observed changes in body composition and preferred substrate utilization by the fish in the present study.

Although the present results reveal major associations between infections with *C. osculatum* and the physiological condition of *G. morhua*, the causality is unclear, and we are still, to some extent, dealing with ‘the chicken or the egg’ dilemma—especially in relation to the strong negative association between the Fulton condition factor and the increasing infection density. In various taxa including fish, the nutritional state of an individual is recognized to impair immune function and thereby increasing the risk of being infected by a pathogen (Gulland, 1992; Chandra, 1997; Johansen *et al*., 1997; Oliva-Teles, 2012). A gradual decrease in the nutritional condition of Eastern Baltic *G. morhua* has occurred since the 1990s, in all likelihood caused by reduced quantity and quality of prey in combination with deteriorating oxygen conditions (Plambech *et al*., 2013; Eero *et al.*, 2015; Casini *et al*., 2016b; Neuenfeldt *et al*., 2020). In an already compromised nutritional state, *G. morhua* may be more susceptible to infection with *C. osculatum*. Notably, and irrespectively of potential causalities, history seems to repeat itself; in the late 1940s, the number of *H. grypus* (i.e. the main final host of *C. osculatum*) in the Baltic Sea was comparable to present days (Harding *et al*., 2007), and at that time, *G. morhua* as now suffered high *C. osculatum* infection rates, with liver lipid content being highly comparable to our study when comparing parasite-free livers with livers infected with *C. osculatum* (Petrushevsky and Shulman, 1955)*.* Furthermore, as in the present study, *G. morhua* infected with *C. osculatum* also had a lower condition as compared with uninfected conspecifics.

Although admittedly speculative, we suggest infections to lead to augmented mortality for the most heavily infected fish (Fig. 7), as also suggested by Horbowy *et al*., 2016. Natural mortality increases for cod in critical condition (Dutil and Lambert, 2000), and the observed very low nutritional condition (Fulton condition factor) combined with low lipid levels in the liver in heavily infected *G. morhua* may thus have fatal consequences for the individual. One could speculate that heavily infected fish exhibit impaired swimming performance where predation on *S. sprattus* may become increasingly challenging, contributing further to the negative association between high infection load and low nutritional condition (Fig. 7). Likewise, cod in poor condition exhibit reduced swimming endurance and cannot achieve as high swimming speeds as more well-conditioned conspecifics (Martínez *et al.*, 2003). Ultimately, this may lead to increased susceptibility towards predation, for example, by the end host of the parasite (Fig. 7).

To fulfil its life cycle, *C. osculatum* depends on its main final host, the oceanic-living mammal *H. grypus*. As such, *H. grypus* not only has the role as a top predator in the Baltic Sea ecosystem, but this marine mammal is also important in structuring part of the community and disease dynamics of *G. morhua* by introducing and maintaining the parasite burden of *C. osculatum*. Oceanic-living mammals are final hosts to all the major groups of parasites in the oceans, many possessing complex life cycles with several intermittent hosts, including invertebrates and fish (McClelland *et al*., 1990). Although marine mammals during periods of time in history have suffered from intense hunting and humans have depleted their populations, in the 20th century, a shift from resource exploitation towards wildlife conservation resulted in the recoveries of many of these populations (Magera *et al*., 2013). The worldwide occurrence of these marine mammals thus stresses the need of further investigations of potential influence of their parasite fauna on community dynamics, not least in relation to the rebuilding of deteriorating fish populations.

## Funding

This work was supported by the European Maritime and Fisheries Fund and The Danish Fisheries Agency (33113-B-16-070 and 33113-B-16-071) and by the European Union’s Horizon 2020 research and innovation program under Grant Agreement No. 773713 (PANDORA).

## Supplementary Material

Electronic_supp_material_Ryberg_M_coaa093
