# Supplementary material for: Physiological condition of Eastern Baltic cod, Gadus morhua, infected with the parasitic nematode Contracaecum osculatum
Source: Conserv Physiol. 2020 Sep 22;8(1):coaa093. doi: 10.1093/conphys/coaa093 (PMC7507771; doi:10.1093/conphys/coaa093)
Supplement: Electronic_supp_material_Ryberg_M_coaa093 [file electronic_supp_material_ryberg_m_coaa093.docx]

## Electronic Supplementary Material for:

Physiological condition of Eastern Baltic cod, *Gadus morhua*, infected with the parasitic nematode *Contracaecum osculatum*

Marie Plambech Ryberg, Peter V. Skov, Niccolò Vendramin, Kurt Buchmann, Anders Nielsen, Jane W. Behrens

1. **Supplementary results**
   1. **Test of collinearity: Variation Inflation Factor (table S1)**

Table S1. Results from the test of collinearity of explanatory variables used in the different analyses. Test of variation inflation factor: $VIF=\left( \frac{1}{1-R_{i}^{2}} \right)$ is used to assess the collinearity between the variables. The tests are only performed once for each batch of fish, and a VIF value is given for each variable. VIF is tested for the models including INF+length, length+total energy and in models including sum worm+ length+liver weight. The latter has been chosen to show that collinearity was low between liver weight and length of fish. Variables with VIF above 10 are defined as critical in relation to collinearity.

| **Batch number of fish** | **Assessment** | **INF+length** | **Sum worm + Length + Liver weight** | **Length +Total energy** |
| --- | --- | --- | --- | --- |
|  |  | VIF | | |
| Batch 1+2+3 | Nutritional condition | 1.05 & 1.05 | 1.3 & 1.8 & 1.5 | - |
| Batch 1 | Aerobic performance  + plasma composition | 1.0 & 1.0 | 1.2 & 1.6 & 1.4 | - |
| Batch 2 | Organ size | 1.0 & 1.0 | 1.5 & 2.5 2.1 | - |
| Batch 3 | Prox com fish  + liver | 1.0 & 1.0 | 1.2 & 2.2 & 2.0 | 1. & 1.0 |

**1.2 Effect of length on estimation if infection density (INF)**

To show that the association between infection density and the different examined parameters is not a result of a length effect, we have tested all the models again, but this time length was kept in the models despite being non-significant in most cases. This analysis revealed that the effect of length did not influence estimates of infection density in any of the cases (Table S2).

Table S2. Additional analysis of the associations between infection density (INF) and the different parameters when length is included in the model, even though length is not significant in most models. In this analysis gender was included in the models where it was significant, however this estimate is not shown here.

| **Assessment** | **Parameter** | **α (INF)** | **μ (length)** | **λ (total energy)** |
| --- | --- | --- | --- | --- |
| **Nutritional condition** | Fulton condition | -0.02(0.003)*** | -0.006(0.002)*** |  |
| **Aerobic performance** | SMR^1^ | -0.01(0.003)** | -0.004(0.004) |  |
| **Organ size** | Pyloric caeca^2^ | -0.02(0.01)* | 2.60(0.36)*** |  |
|  | Intestine^2^ | -0.02(0.01)* | 2.22(0.35)*** |  |
| **Plasma composition** | Total protein^3^ | -0.02(0.005)** | 0.009(0.006) |  |
|  | Globulins^4^ | 0.01(0.003)*** | 0.002(0.004) |  |
|  | A/G | -0.16(0.03)*** | 0.003 (0.04) |  |
|  | Prealbumin^4^ | -0.07(0.02)** | 0.06(0.03)* |  |
|  | Gamma^4^ | 0.02(0.008)* | 0.007(0.009) |  |
|  | Albumin^4^ | -0.15(0.03)*** | 0.005(0.034) |  |
| **Proximate composition of fish** | Total energy^5^ | -0.03(0.01)** | -0.001(0.002) |  |
|  | Water^4^ | 0.003(0.001)* | -3.1^-5(3.1^4) |  |
|  | Protein^5^ | -0.04(0.01)** | -0.002(0.003) |  |
|  | Glycogen^5^ | 0.05(0.01)** | 0.000(0.003) |  |
|  | Ash^4^ | 0.04(0.01)* | 0.003(0.003) |  |
|  | Dry matter^4^ | -0.01(0.006)* | 0.000(0.001) |  |
|  | Protein^5^ |  | 0.000(0.001) | 0.40(0.02)*** |
|  | Glycogen^5^ |  | -0.002(0.002) | -0.38(0.06)*** |
| **Proximate composition of liver** | Lipid^6^ | -0.18(0.04)*** | 0.018(0.009) |  |
|  | Water^4^ | 0.10(0.03)*** | -0.009(0.006) |  |
|  | Dry mat^4^ | -0.10(0.03)*** | 0.011(0.006) |  |
|  | Ash^4^ | 0.10(0.03)** | -0,009(0.007) |  |

- 1. **Model validation plots**

**
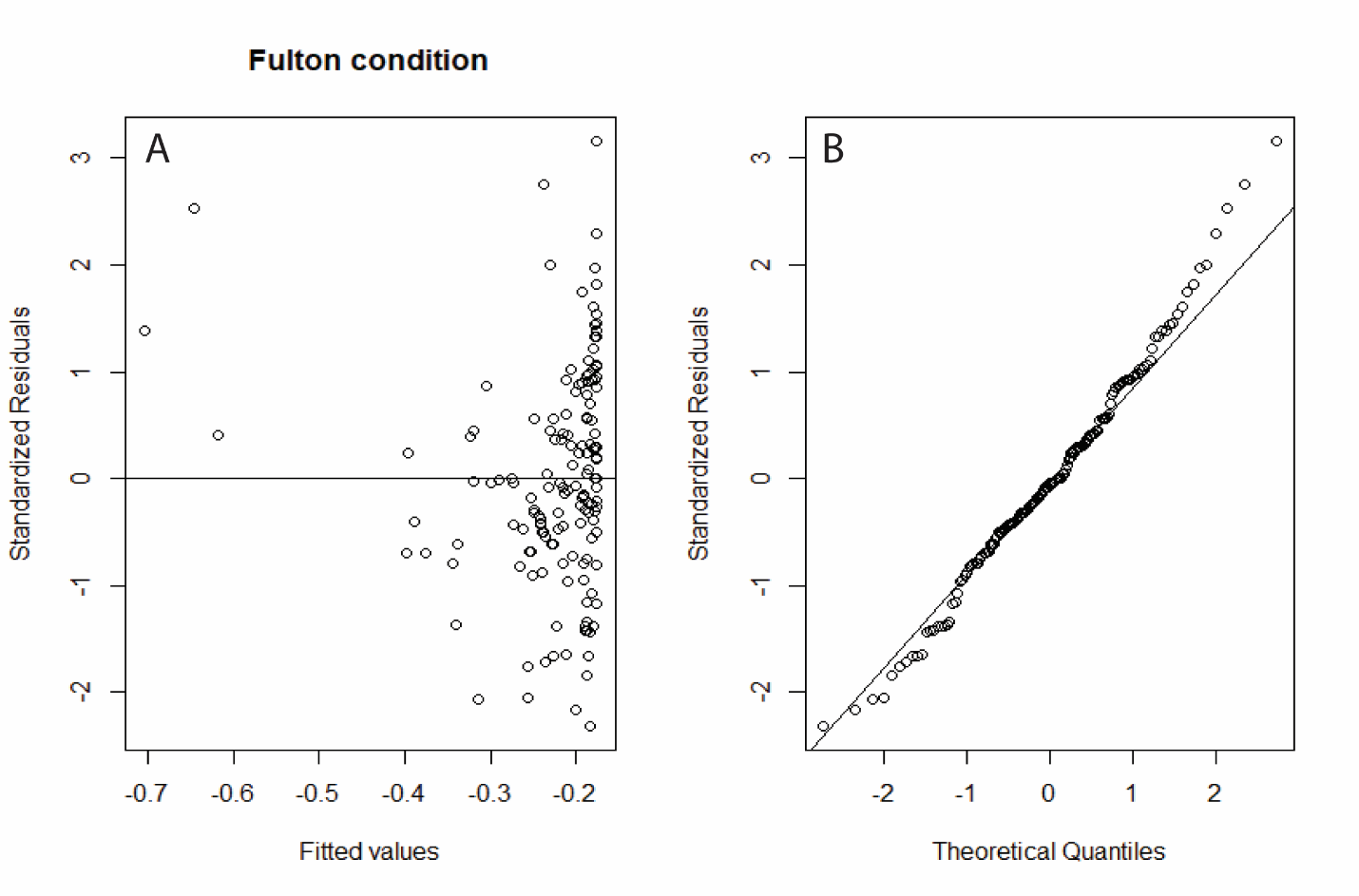
**Fulton condition factor is calculated based on data compiled from all three batches of fish (i.e. all fish used in this study, n=152) (Fig S1). Examination of aerobic performance and plasma composition was performed on the same batch of fish (Figs. S2-S5). The study where changes in organ size was investigated involved fish from the second batch which was only used in in this context (Fig S6). Analysis of composition of the fish and the liver was based on a third batch of fish and figure S7-S12 are therefore showing the same fish ID’s.

Figure S1. Model diagnostic plots for the final model of Fulton condition factor showing A) standardized residuals and fitted values and B) Q-Q plot for normal distribution of residuals.


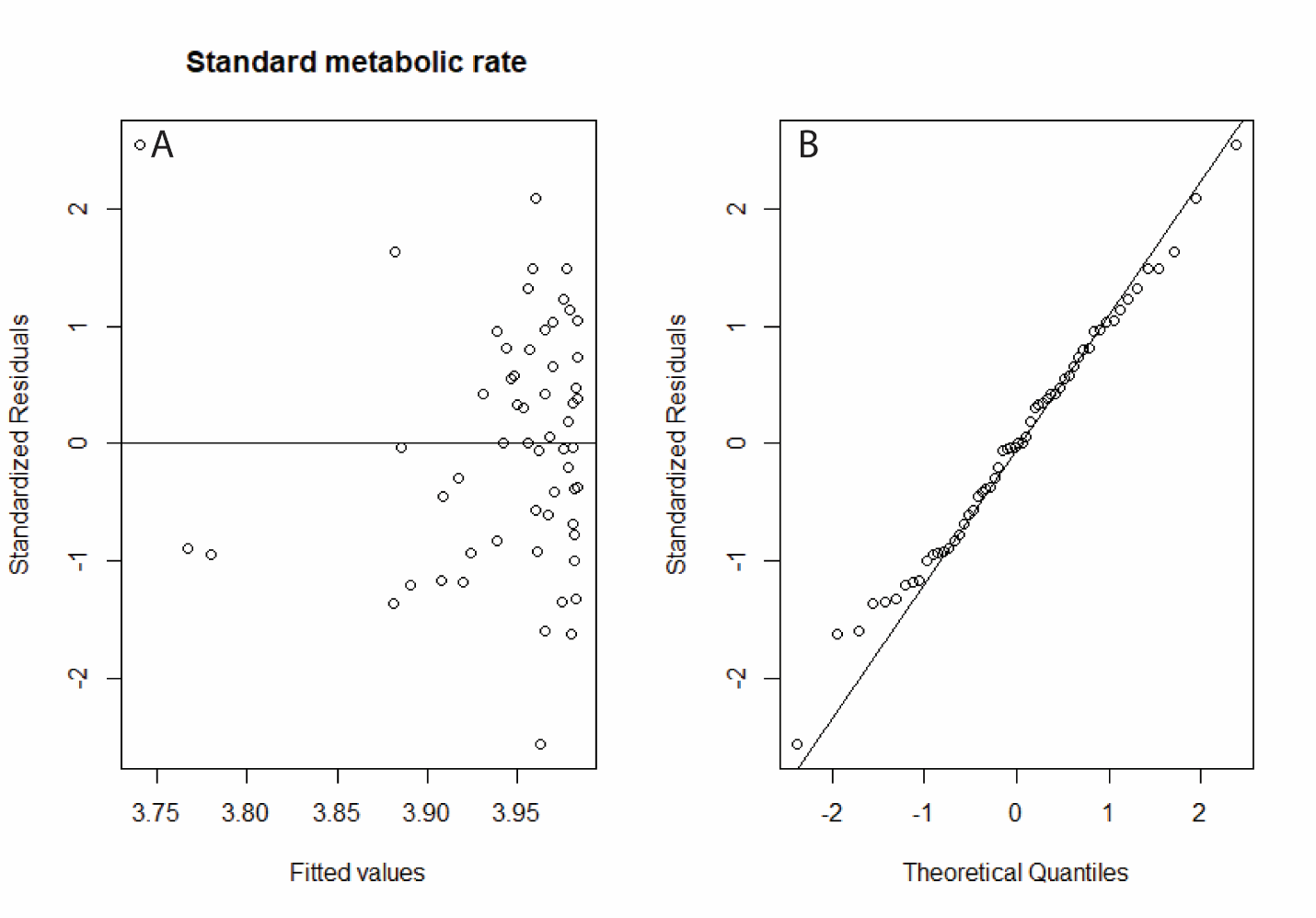


Figure S2. Model diagnostic plots for the final model of standard metabolic rate (SMR) showing A) standardized residuals and fitted values and B) Q-Q plot for normal distribution of residuals.


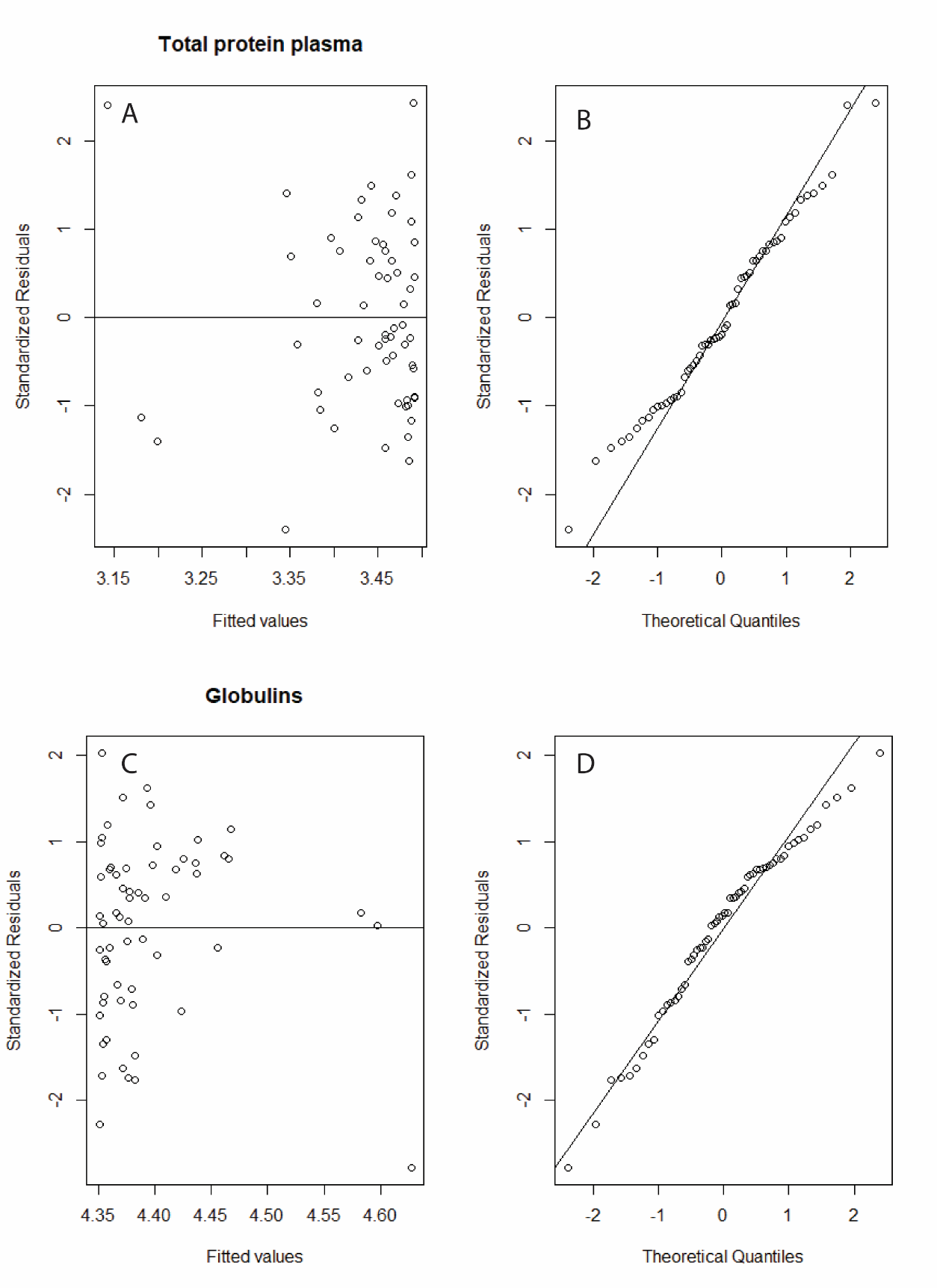
Figure S3. Model diagnostic plots for the final model of total protein (A+B) and globulins (C+D) in blood showing A+C) standardized residuals and fitted values and B+D) Q-Q plot for normal distribution of residuals.


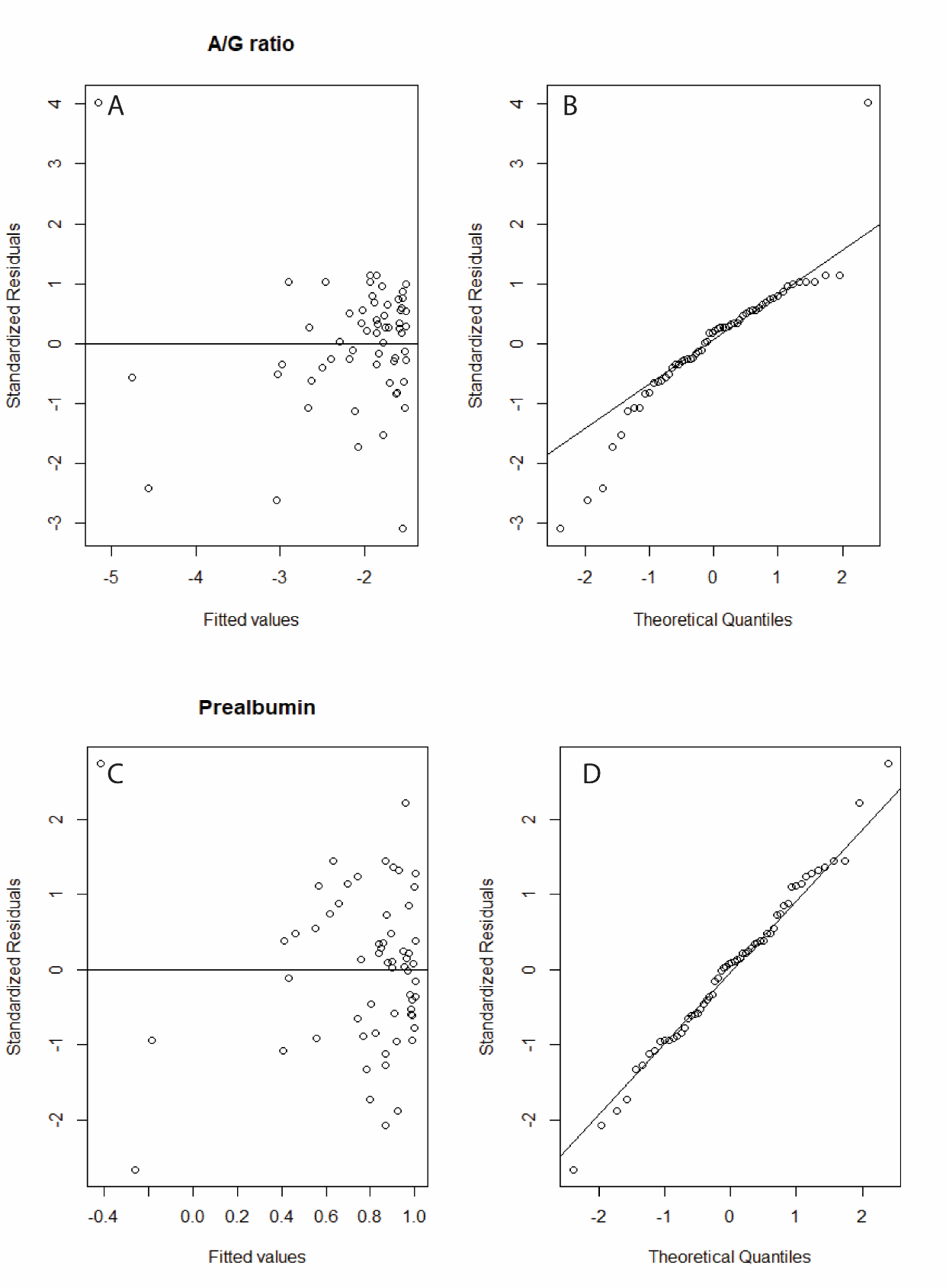
Figure S4. Model diagnostic plots for the final model of A/G ratio (A+B) and prealbumin (C+D) showing A+C) standardized residuals and fitted values and B+D) Q-Q plot for normal distribution of residuals.


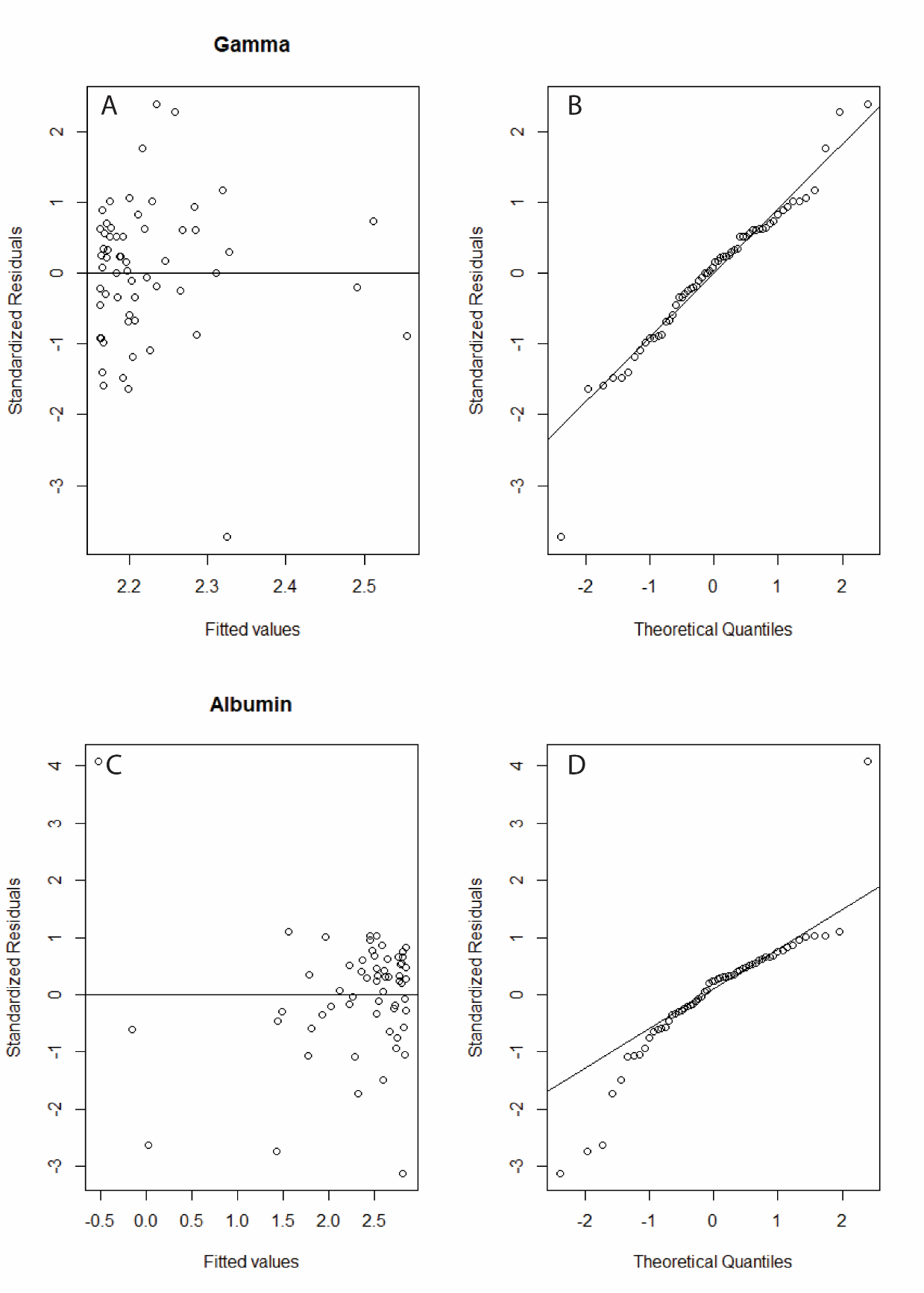
Figure S5. Model diagnostic plots for the final model of gamma (A+B) and albumin (C+D) showing A+C) standardized residuals and fitted values and B+D) Q-Q plot for normal distribution of residuals.


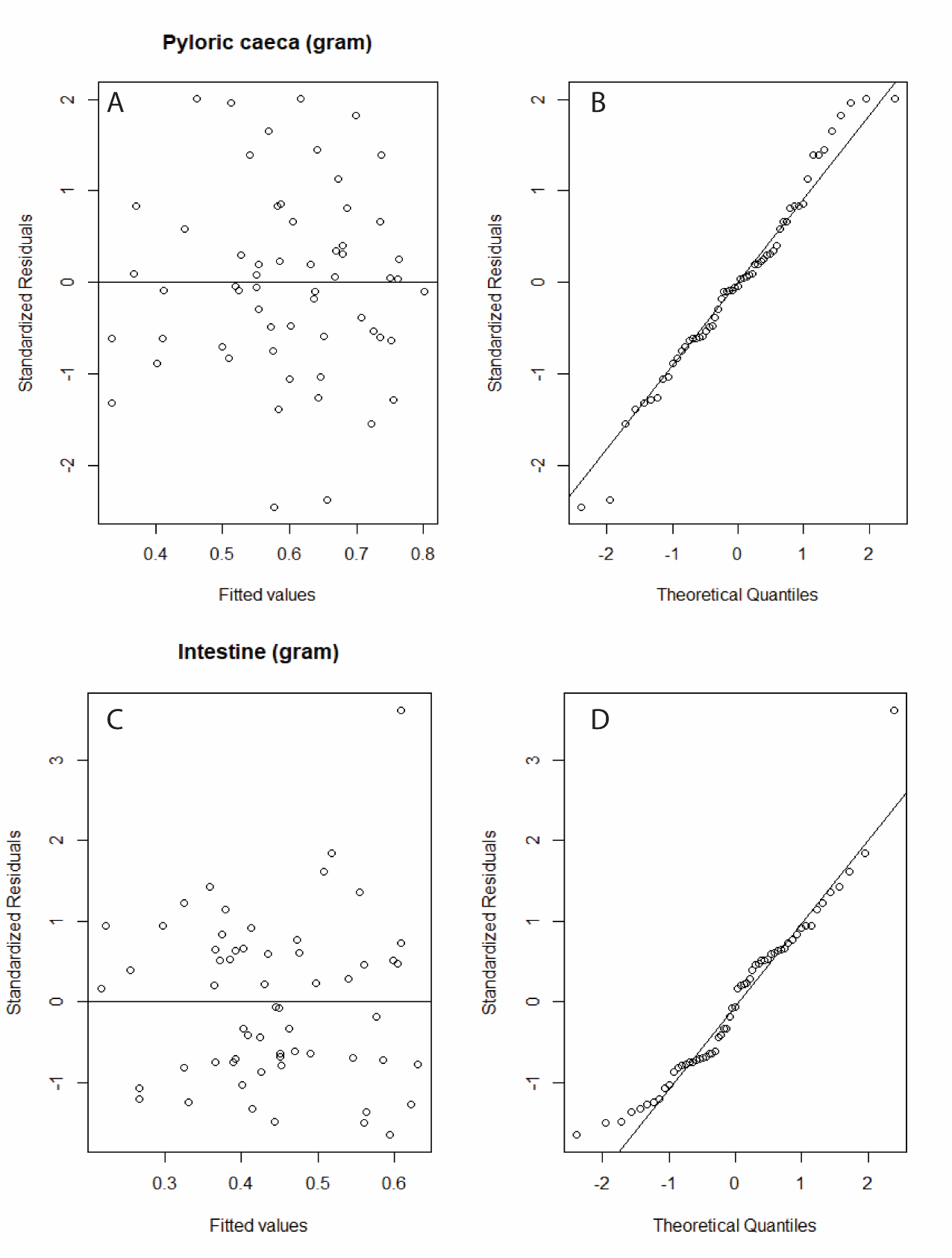
Figure S6. Model diagnostic plots for the final model of pyloric caeca (A+B) and intestine (C+D) showing A+C) standardized residuals and fitted values and B+D) Q-Q plot for normal distribution of residuals.


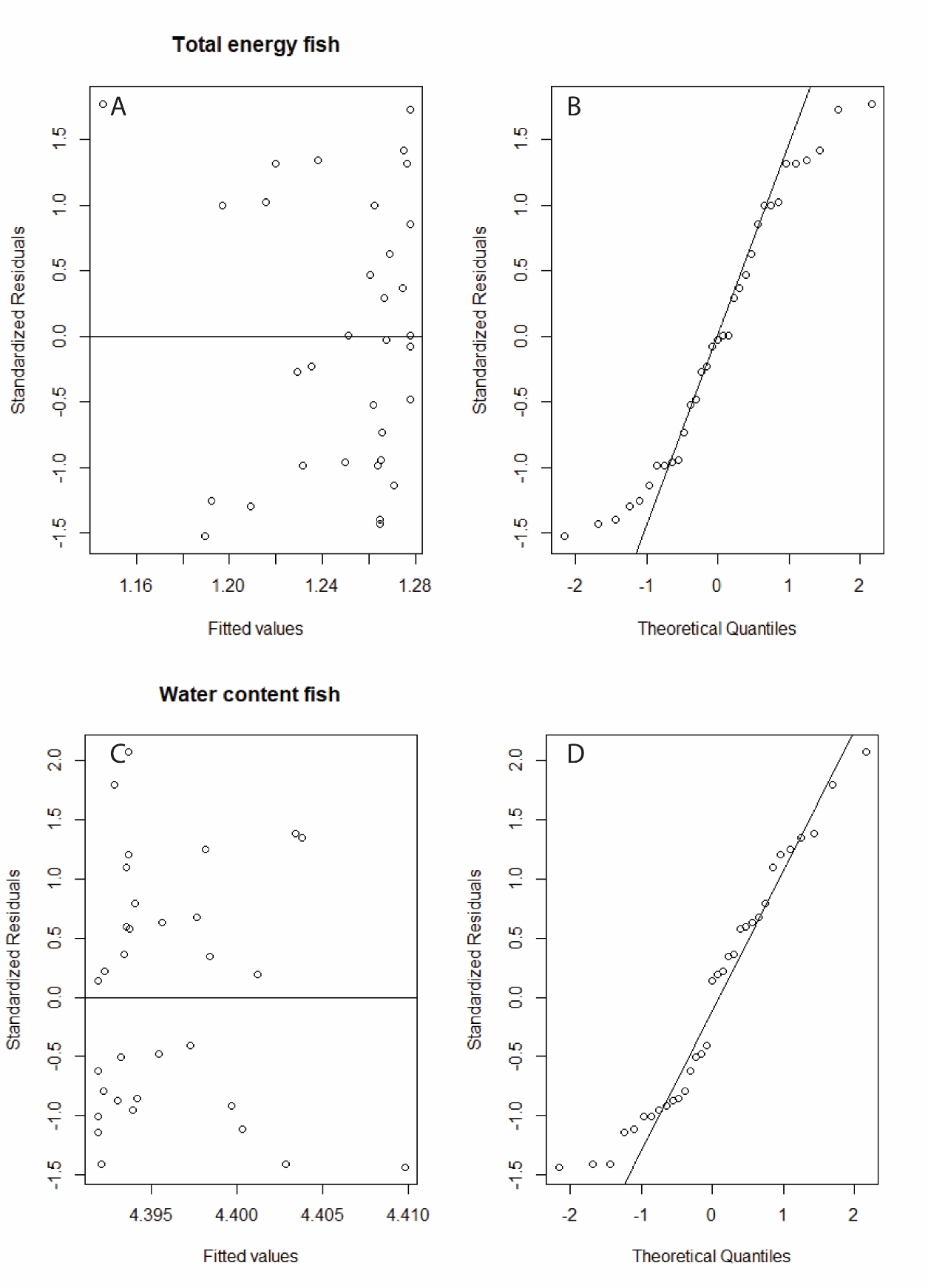
Figure S7. Model diagnostic plots for the final model of total energy (A+B) and water of fish (C+D) showing A+C) standardized residuals and fitted values and B+D) Q-Q plot for normal distribution of residuals.


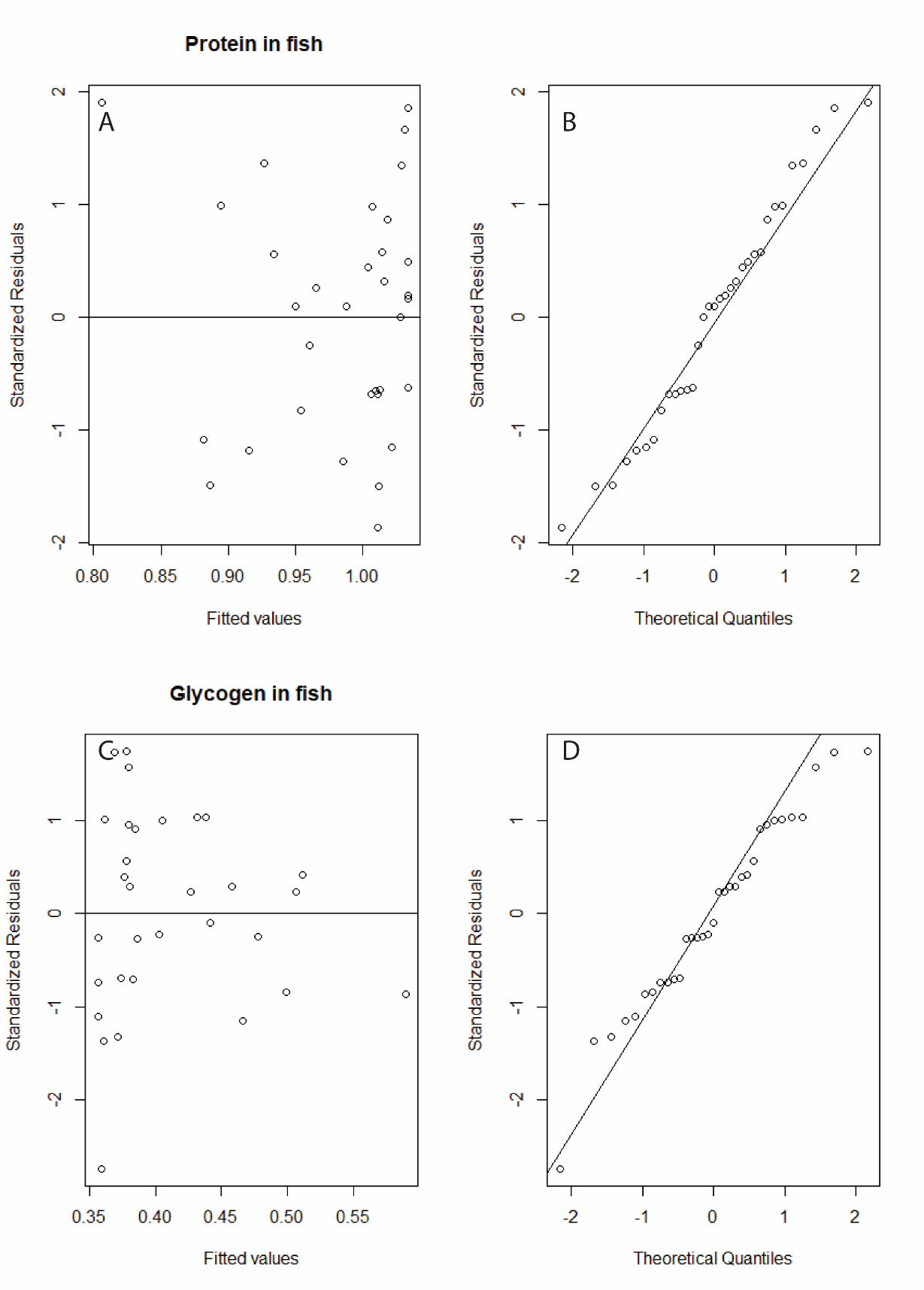
Figure S8. Model diagnostic plots for the final model of protein (A+B) and glycogen in fish (C+D) showing A+C) standardized residuals and fitted values and B+D) Q-Q plot for normal distribution of residuals.


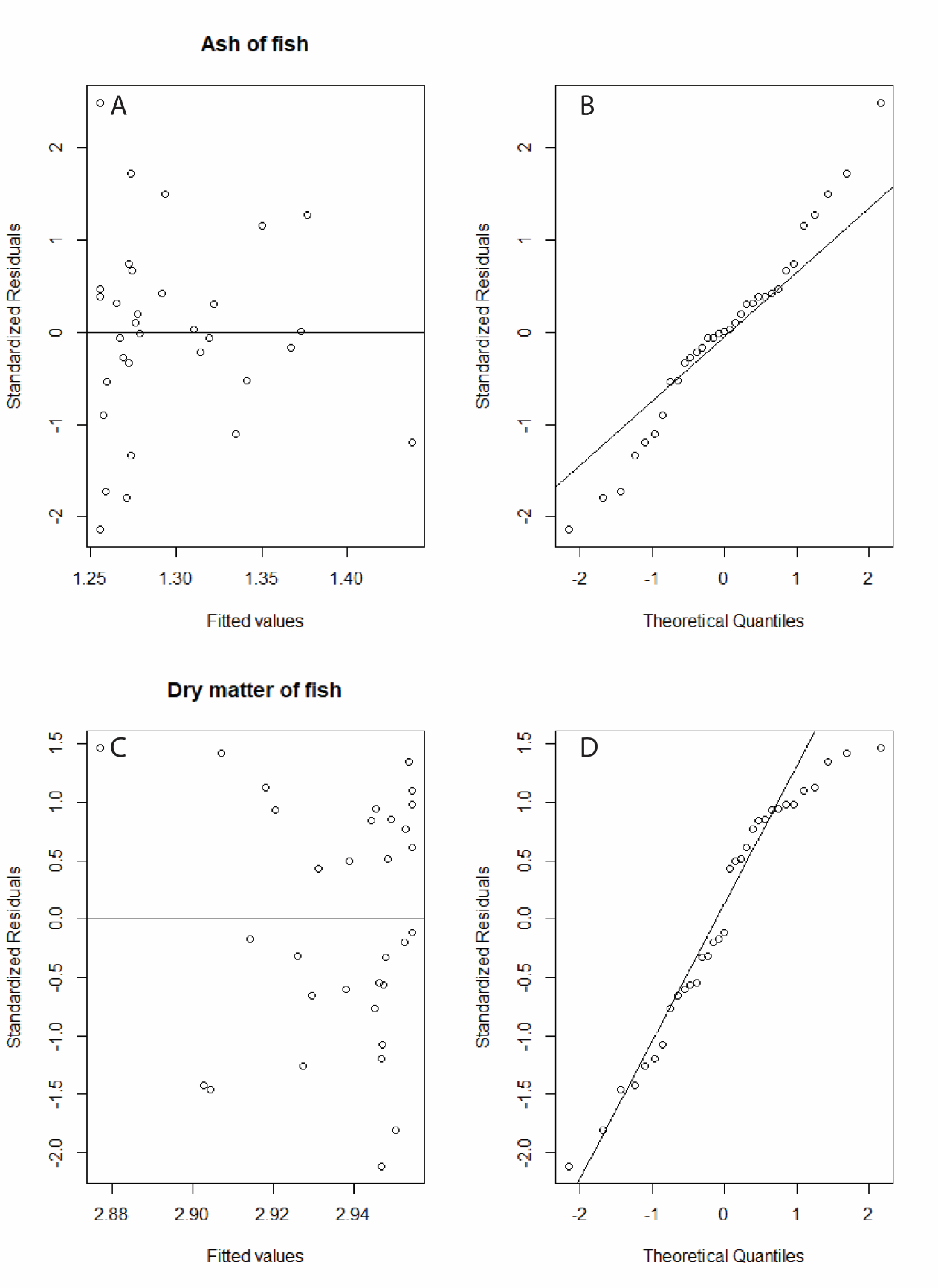
Figure S9. Model diagnostic plots for the final model of Ash (A+B) and dry matter of fish (C+D) showing A+C) standardized residuals and fitted values and B+D) Q-Q plot for normal distribution of residuals.


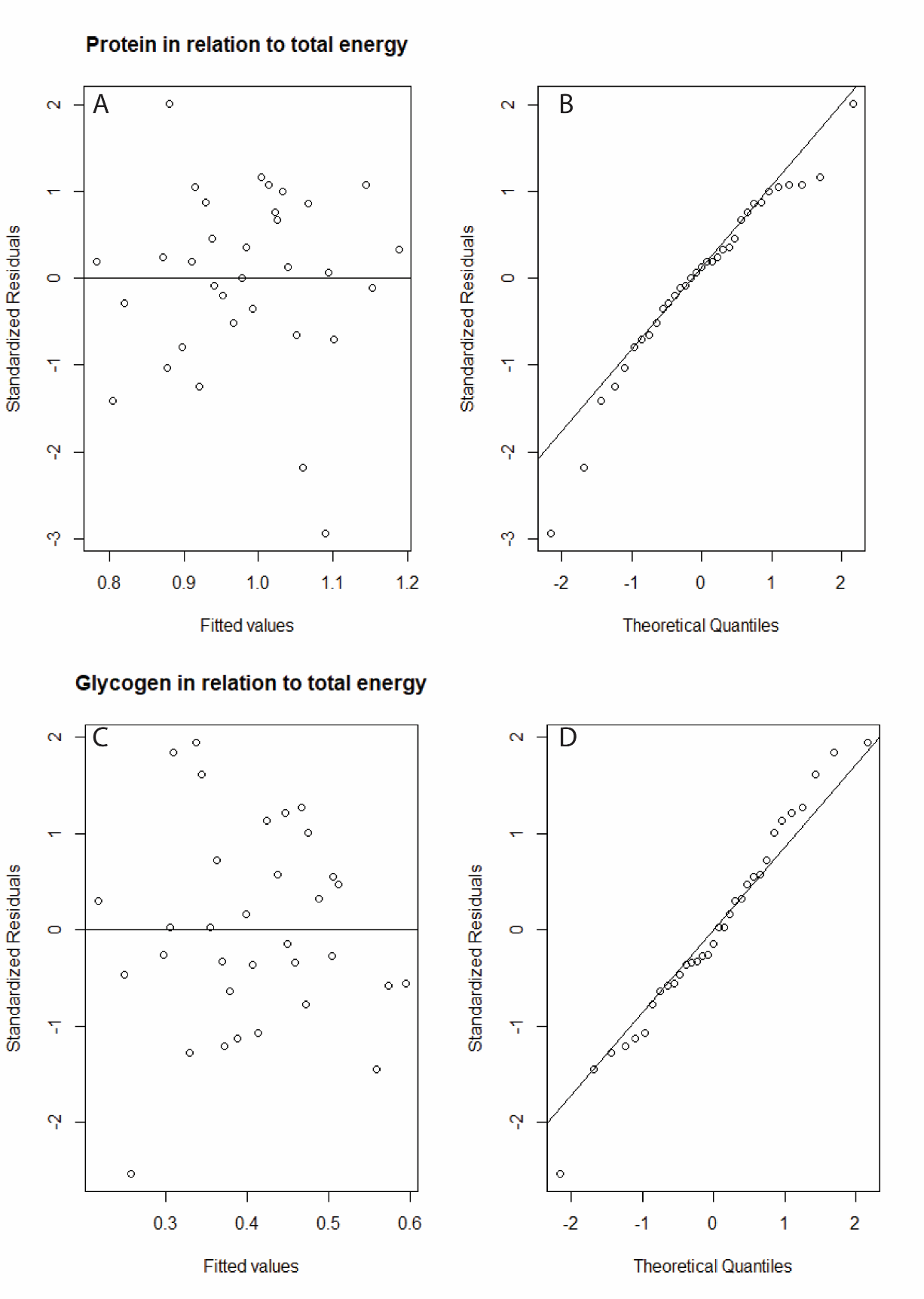
Figure S10. Model diagnostic plots for the final model of protein (A+B) and glycogen versus total energy (C+D) showing A+C) standardized residuals and fitted values and B+D) Q-Q plot for normal distribution of residuals.


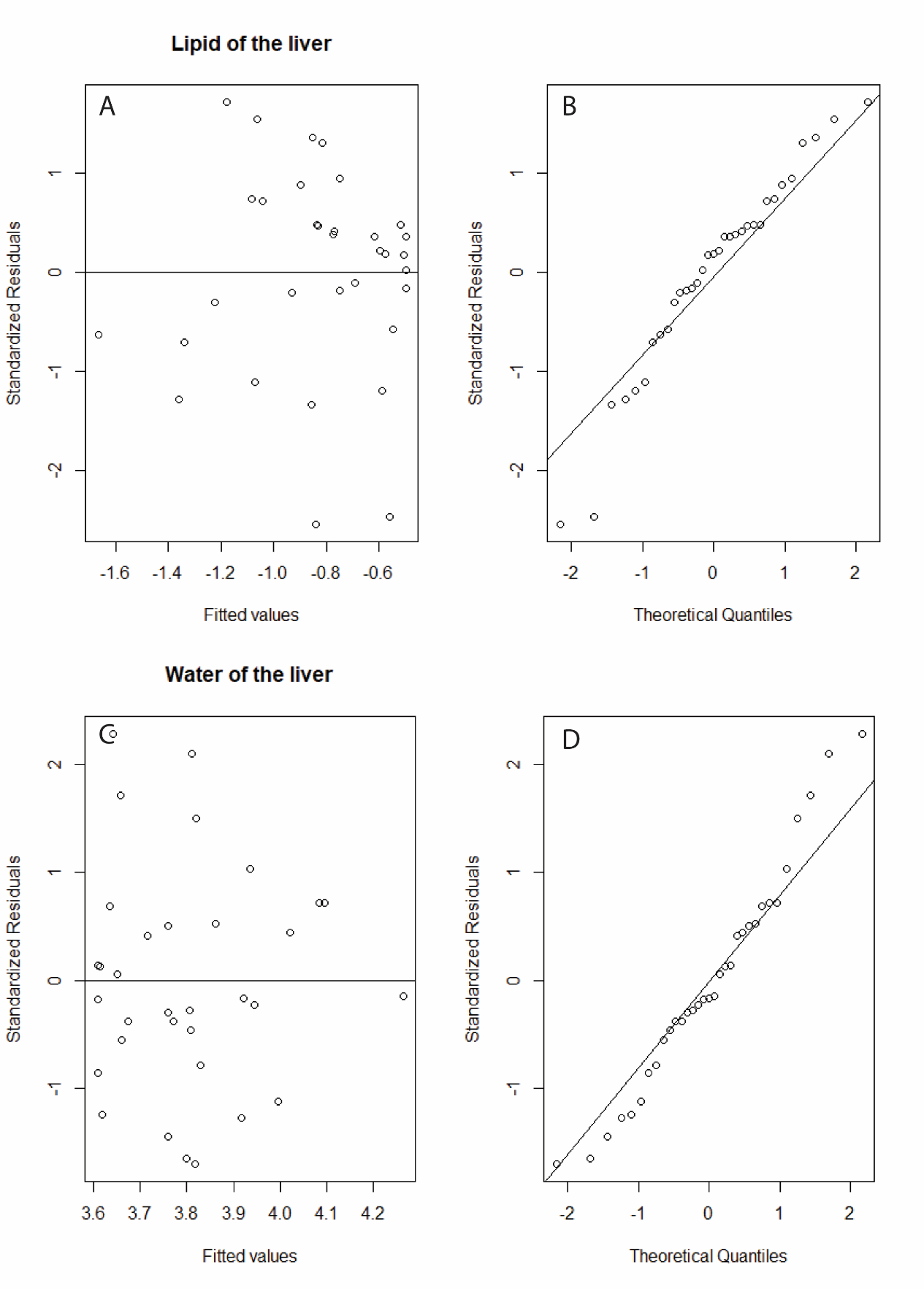
Figure S11. Model diagnostic plots for the final model of lipid (A+B) and water of liver (C+D) showing A+C) standardized residuals and fitted values and B+D) Q-Q plot for normal distribution of residuals.


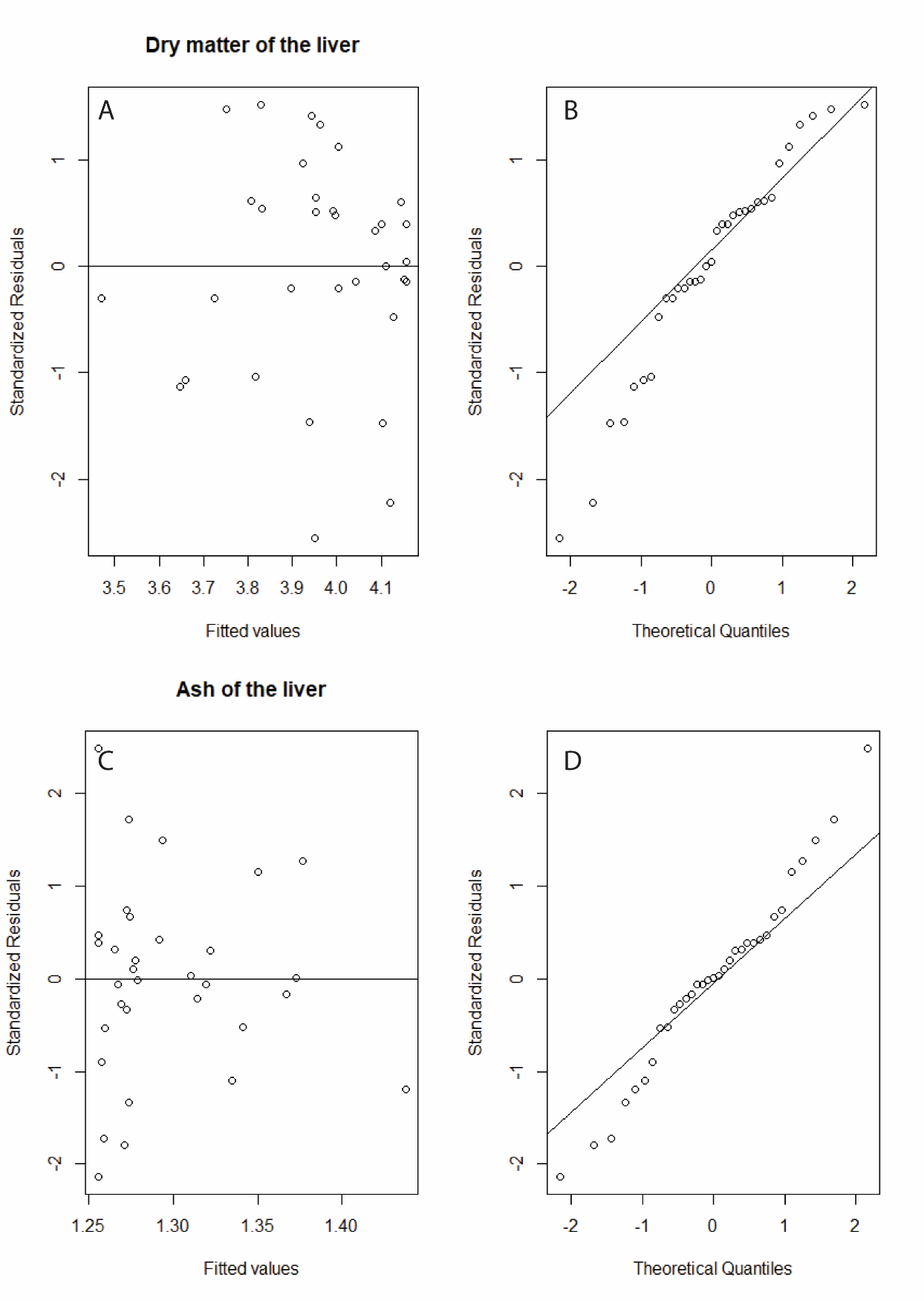
Figure S12. Model diagnostic plots for the final model of dry matter (A+B) and ash of liver (C+D) showing A+C) standardized residuals and fitted values and B+D) Q-Q plot for normal distribution of residuals.
